# Identification of functionally important domains of human cytomegalovirus gO that act after trimer binding to receptors

**DOI:** 10.1371/journal.ppat.1010452

**Published:** 2022-04-22

**Authors:** Andrea Chin, Jing Liu, Theodore Jardetzky, David C. Johnson, Adam Vanarsdall

**Affiliations:** 1 Department of Molecular Microbiology & Immunology, Oregon Health & Science University, Portland, Oregon, United States of America; 2 Department of Structural Biology, Stanford University School of Medicine, Stanford, California, United States of America; University of Alabama at Birmingham, UNITED STATES

## Abstract

Human cytomegalovirus (HCMV) entry involves trimer (gH/gL/gO) that interacts with PDGFRα in fibroblasts. Entry into epithelial and endothelial cells requires trimer, which binds unidentified receptors, and pentamer (gH/gL/UL128-131), which binds neuropilin-2. To identify functionally important domains in trimer, we screened an overlapping 20-mer gO peptide library and identified two sets of peptides: 19/20 (a.a. 235–267) and 32/33 (a.a. 404–436) that could block virus entry. Soluble trimer containing wild type gO blocked HCMV entry, whereas soluble trimers with the 19/20 or 32/33 sequences mutated did not block entry. Interestingly, the mutant trimers retained the capacity to bind to cellular receptors including PDGFRα. Peptide 19/20 and 32/33 sequences formed a lobe extending from the surface of gO and an adjacent concave structure, respectively. Neither of these sets of sequences contacted PDGFRα. Instead, our data support a model in which the 19/20 and 32/33 trimer sequences function downstream of receptor binding, e.g. trafficking of HCMV into endosomes or binding to gB for entry fusion. We also screened for peptides that bound antibodies (Abs) in human sera, observing that peptides 20 and 26 bound Abs. These peptides engendered neutralizing Abs (NAbs) after immunization of rabbits and could pull out NAbs from human sera. Peptides 20 and 26 sequences represent the first NAb epitopes identified in trimer. These studies describe two important surfaces on gO defined by: i) peptides 19/20 and 32/33, which apparently act downstream of receptor binding and ii) peptide 26 that interacts with PDGFRα. Both these surfaces are targets of NAbs.

## Author summary

Human cytomegalovirus (HCMV) infects 80% of the world population, causing severe morbidity and mortality in transplant patients and can be transmitted to the developing fetus leading to severe neurological defects. The current anti-viral agents used to treat HCMV are not very effective as viruses can develop resistance and there is no licensed HCMV vaccine available. Recently, there has been intense interest in the HCMV envelope glycoproteins involved in entry as a component of vaccines. One glycoprotein complex, the gH/gL/gO trimer is especially intriguing as it is required for infection of extracellular virus in all cell types. Here, we identify domains in the trimer that have an essential function in entry downstream of receptor binding and are also epitopes recognized by naturally induced neutralizing antibodies. These results will have implications for advancing the efforts to develop novel HCMV therapeutics.

## Introduction

Human cytomegalovirus (HCMV) is a ubiquitous β-herpesvirus that establishes lifelong persistent or latent infection and is typically benign in immunocompetent individuals [1–4]. However, HCMV infections are a major problem in transplant patients, contributing to viral disease and graft failure [5–7]. Further, HCMV is the most common viral infection in the developing fetus, affecting ~0.5% of newborns [3,8–10]. Congenital HCMV infections lead to damage to the developing nervous system, most often producing hearing loss, which accounts for 25% of children with sensorineural hearing loss in the United States [11]. There is an urgent need for HCMV vaccines, especially to protect babies. The viral entry glycoproteins are important targets of host immunity and, thus, may be crucial in the development of viral vaccines.

Acute HCMV infections frequently involve dissemination throughout the body, so that many organs and cell types are infected: epithelial cells, fibroblasts, smooth-muscle cells, monocyte-macrophages (M/Ms), and endothelial cells among others [12]. Tropism of HCMV depends upon the utilization of different viral entry machineries (reviewed in [13–17]. The entry of β-herpesviruses into cells shares many similarities with α-herpesviruses and γ-herpesviruses that all use gB fusion proteins and gH/gL proteins to trigger gB for fusion. However, unlike α-herpesviruses, β-herpesvirus assemble several complexes of gH/gL including gH/gL/UL128-131 denoted the pentamer [15,18], gH/gL/gO called the trimer [19,20] and gB/gH/gL [21,22]. These different gH/gL complexes play separate roles in meditating virus entry into different cell types. The pentamer is required for entry into epithelial and endothelial cells and monocyte-macrophages, but is not required for entry into fibroblasts [23,24].

Our early studies involving a HCMV gO-null mutant demonstrated that trimer (gH/gL/gO) is essential for entry of extracellular virus particles (here the word entry refers to extracellular particles) into all the cells tested: epithelial and endothelial cells and also fibroblasts, though this mutant could spread cell-to-cell [25]. Interference studies suggested that trimer binds to saturable cellular receptors that mediate HCMV entry [26]. Studies of HCMV entry into fibroblasts demonstrated that trimer binds with high affinity to platelet derived growth factor receptor-α (PDGFRα) to mediate entry into the cells [27–30]. However, there is good evidence that entry of HCMV into epithelial and endothelial cells does not involve PDGFRα [28,29,31,32]. Supporting this conclusion, soluble trimer could bind to the surfaces of epithelial cells and block virus entry, and this binding did not involve PDGFRα [33]. These studies support a model in which PDGFRα acts as receptor for entry into fibroblasts, but entry into epithelial and endothelial cells involves unidentified trimer receptors, as well as the pentamer receptors: neuropilin-2 (NRP-2) and OR14I1 [34,35]. Two other trimer receptors were identified in screens for trimer binding proteins: TGFbRIII and NRG2, however trimer bound with extremely low affinity to NRG2 (too low to be biologically important) and was not characterized further. TGFbRIII was not functionally important for HCMV entry [35,36]. Entry into epithelial cells involves the unidentified trimer receptors followed by macropinocytosis and pentamer-mediated exit from endosomes [31,33]. At present, it is not yet clear how trimer binding to PDGFRα or pentamer binding to receptors Nrp-2 or OR14I1 leads to activation of gB for entry fusion [34,35]

There are detailed structures of the HCMV pentamer, pentamer with neutralizing Abs (NAbs) bound, and pentamer bound to NRP-2 [15,35,37,38]. By contrast, until very recently there was only a low-resolution structure of the trimer [37] and little information on where gO-specific Abs bind. Sinzger and colleagues described functionally important domains in gO in terms of binding PDGFRα and entry into cells. Their mutational screen of conserved gO sequences described a mutant form of gO involving a.a. 249–254 that exhibited highly reduced entry of cell-free virus, as well as mutations nearer the N-terminus of gO that reduced entry [32,39]. Recently, while this manuscript was being written, two detailed structures of the HCMV trimer in contact with PDGFRα were reported, including one from our group [36,40].

Studies of antibodies (Abs) specific for pentamer and trimer have also produced evidence for their functional significances. Pentamer-specific monoclonal Abs (MAbs), polyclonal sera from rabbits, and Abs from human sera are strongly neutralizing [41–46]. There was also evidence that transmission of HCMV from mothers to babies in utero was prevented by pentamer-specific Abs [47]. We recently characterized antibodies present in sera from human transplant patients and mothers for neutralizing Abs that reacted with gH/gL, pentamer and trimer [48]. Pentamer-specific Abs tended to be higher in titer, but trimer-specific Abs were often present at comparable neutralizing titers and gH/gL-specific Abs rarely neutralized HCMV. There was no correlation between neutralizing Abs (NAbs) and virus transmission from mothers to babies. Importantly, trimer-and pentamer-specific Abs acted in a synergistic fashion to neutralize HCMV [48]. Given that trimer is essential for HCMV entry into all cell types and pentamer is important for entry into most cell types, it makes ample sense that both proteins are targets of neutralizing Abs.

To identify and characterize functionally important domains of the gO polypeptide, we screened a library of overlapping 20 amino acids (a.a.) peptides for their capacity to block HCMV entry into fibroblasts and epithelial cells. Two sets of overlapping peptides including those that contained sequences: i) F_235_-K_267_ and ii) S_404_-P_436_ inhibited HCMV entry into both fibroblasts and epithelial cells. Soluble trimer proteins containing mutations of 2 or 5 a.a. in these sequences, failed to block virus entry into fibroblasts and epithelial cells, whereas wild type trimer inhibited entry. The mutant trimers bound to both types of cells and also bound to PDGFRα. The peptides were also used to screen for the presence of NAbs in human sera. One of the peptides that blocked virus entry F_235_-K_267_ also contained sequences recognized by NAbs and a second peptide containing sequences L_326_-C_345_ was recognized by NAbs. Rabbits immunized with these peptides produced HCMV-neutralizing Abs. Thus, these studies defined three linear domains in gO that are important for how the trimer functions in entry and recognition by NAbs. Importantly, 2 of these gO protein sequences (peptides 19/20 and 32/33) were not obviously involved in binding to PDGFRα or trimer receptors on epithelial cells.

## Results

### HCMV gO peptides that inhibit virus entry into fibroblasts and epithelial cells

**Fig 1:**
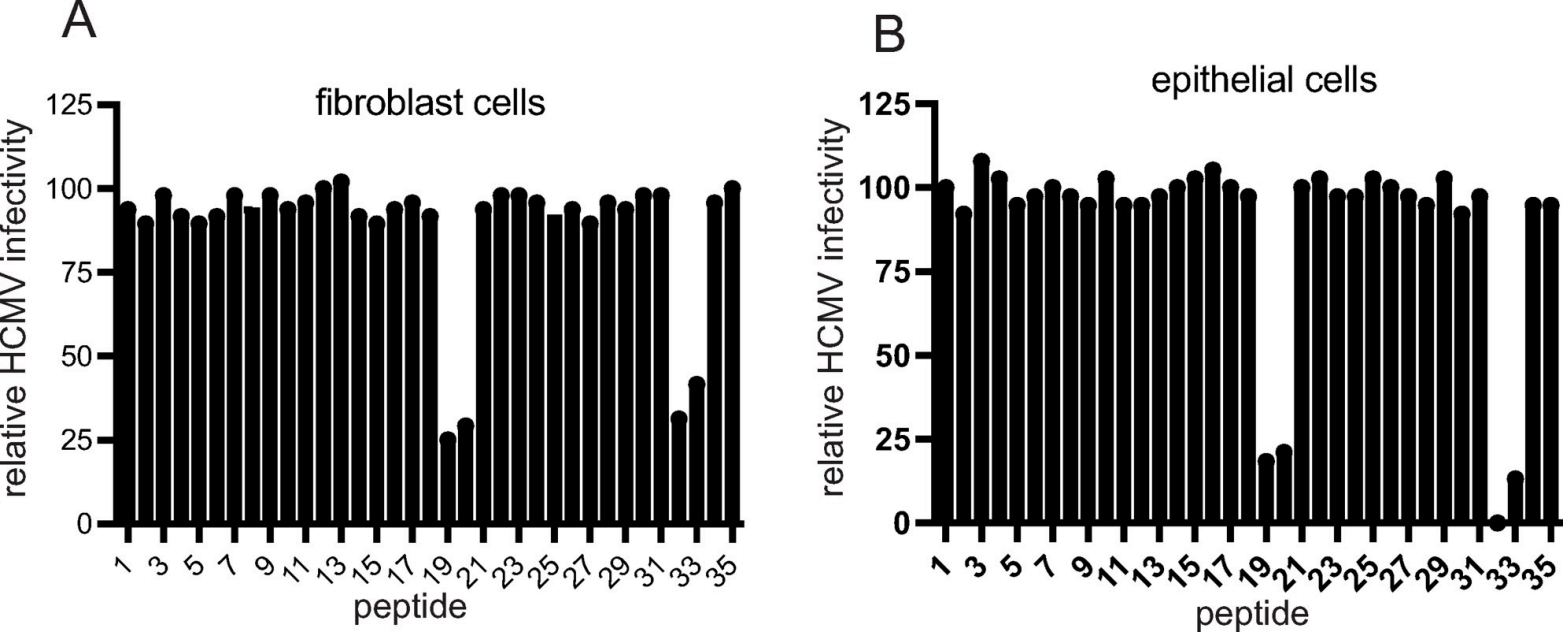
Peptide inhibition of HCMV entry into of fibroblasts and epithelial cells. Individual peptides from an overlapping peptide library were incubated with fibroblasts (A) or ARPE-19 epithelial cell (B) monolayers using 100 μM of each of the peptides in media lacking serum for 1 h at 4° C. Subsequently HCMV BADrUL131, which expresses GFP, was added to the cells in the presence of the peptides and incubated for 1 h at 4° C. The cells were then shifted to 37° C for 2 h. The cells were then washed and incubated in fresh growth media containing of peptides at 37° C for 24 h. The level of virus entry was determined by assessing GFP expression by fluorescent microscopy and comparing to control conditions lacking peptides and expressed as percent infection relative to the control.

Previously, we demonstrated that a soluble form of the HCMV trimer blocked HCMV entry into epithelial cells and fibroblasts [33]. To define functionally important domains in gO involved in entry, we obtained a library of 20 amino acid (a.a.) peptides spanning the entire HCMV TR gO polypeptide with 7 a.a. overlaps (S1 Table). It is important to note that previous studies of gO involved numbering amino acids beginning with the initiating methionine [32,39,49,50] and to reduce confusion we numbered gO residues beginning with the initiating methionine. An initial screen involved testing whether peptides would inhibit entry of HCMV into fibroblasts and epithelial cells. In these studies, we used HCMV BADrUL131, a virus derived from strain AD169 that expresses GFP and expresses the pentamer and can infect epithelial and endothelial cells [51]. Fibroblast or epithelial cells were incubated with individual peptides followed by incubation with BADrUL131 for 2 h and then the number of GFP positive cells enumerated 24 h later. Two sets of overlapping peptides: i) 19 (a.a. 235–254) and 20 (a.a. 248–267) and ii) 32 (a.a. 404–423) and 33 (a.a. 417–436) inhibited virus entry and this inhibition of virus entry was observed with both fibroblasts and epithelial cells (Fig 1). Both peptides 19 and 20 contain the 7 a.a. sequence: N_248_TMRKLK_254_. Both Peptides 32 and 33 contain the sequence: D_417_YLDSLL_423_.

**Fig 2:**
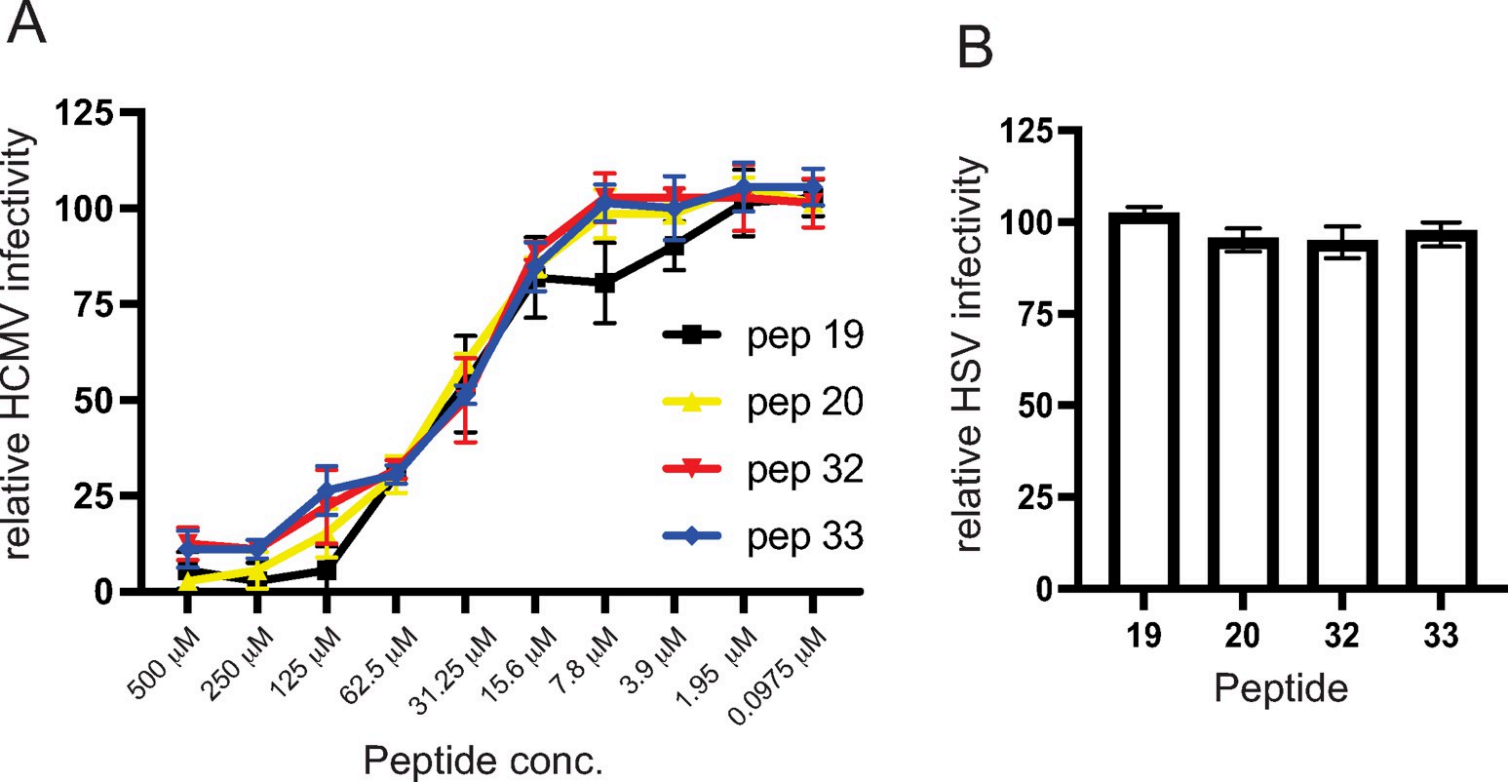
Dose responses of gO peptides and HSV control experiment. (A) gO peptides 19, 20, 32, and 33 were diluted then incubated with ARPE-19 epithelial cell monolayers using a range of concentrations for 1 h at 4° C and then HCMV BADrUL131 was added to the cells in the presence of peptides and incubated and additional 1 h at 4° C. The cells were then shifted to o 37° C for 2h. The cells were washed then incubated in fresh growth media supplemented with peptides at 37° C for 24 h. The level of virus entry was determined described in Fig 1. Data was collected from 3 separate wells for each condition. (B) gO peptides 19, 20, 32, and 33 (each at 100 μM) were incubated with epithelial cells then incubated with HSV expressing a GFP-tagged capsid protein (VP26), as described above for HCMV entry assays. The cells were then washed and incubated in fresh growth media containing peptides at 37° C for 24 h and virus entry assessed by determining the number of GFP expressing cells and comparing to control conditions lacking peptides. Data was collected from 3 separate wells.

We characterized the inhibition of HCMV entry into epithelial cells using different doses of peptides 19, 20, 32, and 33. All peptides inhibited HCMV entry by 88–95% at 500 μM (Fig 2A) and there was half maximal inhibition of the peptides with approximately 35 μM of each of the four peptides (Fig 2A). Peptide 19 produced lower inhibition compared with the other peptides at 7.8 μM. To rule out toxic or non-specific effects of these peptides, we tested whether these peptides reduced entry and gene expression of an HSV VP26-GFP recombinant virus. No reduction in HSV entry and GFP-VP26 expression was observed with peptides 19, 20, 32, and 33 after 24 h (Fig 2B). In addition, there was no non-specific inhibition of virus entry using a control peptide (peptide # 21) that was not identified in the initial screen under these conditions (S1 Fig).

### HCMV trimers containing gO mutations fail to block HCMV entry

**Fig 3:**
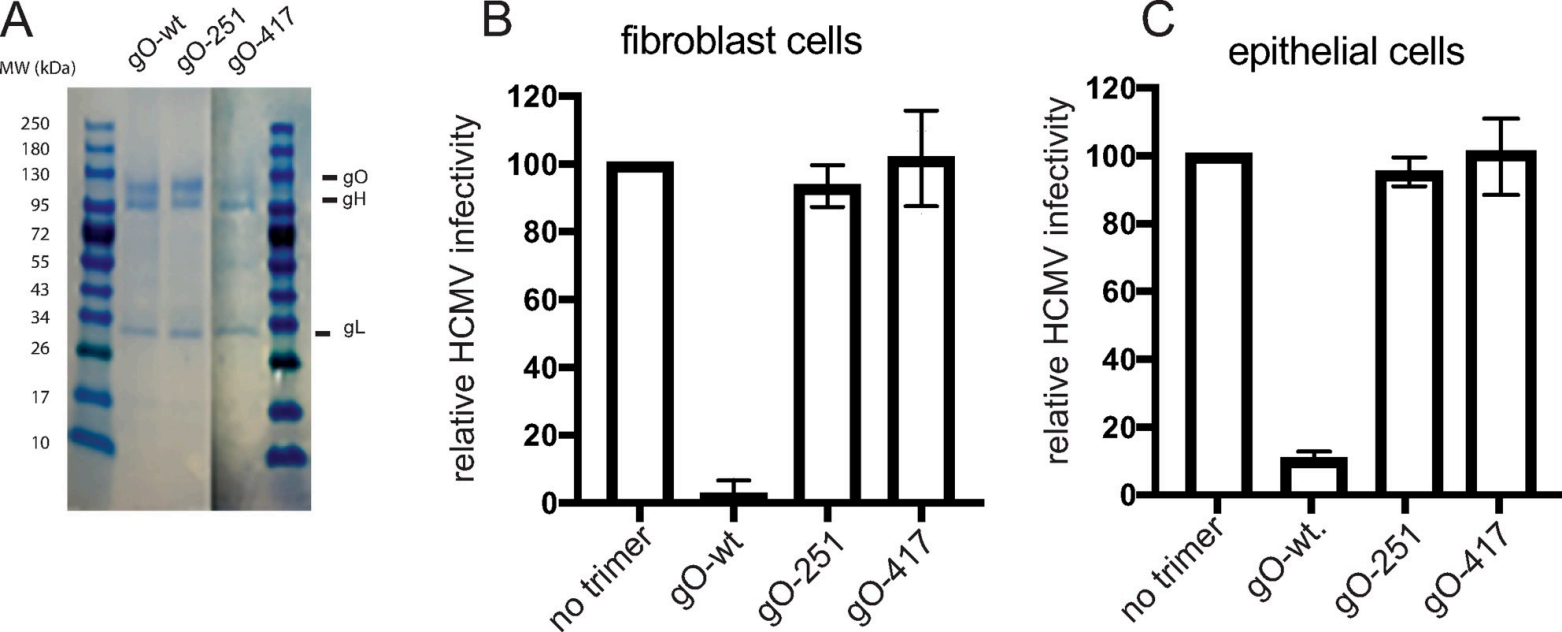
Trimer molecules containing mutant forms of gO fail to block HCMV entry. (A) Trimer molecules purified from 293 6E cells were loaded and separated by SDS-PAGE under reducing conditions followed by staining the gels with GelCode Blue (Thermo). Fibroblasts cells (panel A) were either left untreated (no trimer) or incubated with trimer that contained: gO-wt (5 μg/ml), gO-251 (5 μg/ml), or gO-417 (10 μg/ml). ARPE-19 epithelial cells (panel B) were either left untreated (no trimer) or incubated with trimer that contained: gO-wt (25 μg/ml), gO-251(25 μg/ml), or gO-417 (50 μg/ml). in both cases, soluble proteins were incubated with cells for 1 h at 4°C then HCMV BAD*r*UL131 that expresses GFP was added to the cells in the presence of the trimers and incubated for an additional 1 h at 4°C. The cells were then shifted to 37° C and incubated for an additional 2 h (with trimer present) then the virus inoculum was removed, the cells washed, and then incubated in fresh growth media at 37° C for 24 h. The level of virus entry was determined by assessing GFP expression comparing to the no trimer conditions. The data was determined from three separate wells for each condition.

To test these peptide sequences in the context of trimer proteins, we constructed mutant forms of the gO protein in the overlap regions of peptides 19/20, and 32/33. One mutant gO protein, *denoted gO-251*, contained alanine substitutions in the overlapping sequences of peptides 19/20: R_251_KLKRK ➔AALAAA. The second mutant, *denoted gO-417*, contained alanine substitutions in the overlapping sequences in peptides 32/33: D_417_YLD➔ AYLA. These mutants were co-expressed with a soluble form of gH (lacking the transmembrane domain and containing a poly-histidine tag) and gL in 293-6E cells and the secreted trimer proteins were purified from culture supernatants using nickel agarose, as described [33]. gO-251 assembled onto gH/gL well, there was similar levels of gO associated with gH/gL that was pulled down using the poly-histidine tag on gH (Fig 3A). However, there was less gO-417 in trimer preparations, compared with that observed in trimers containing gO-251 and gO-wt. Nevertheless, the gO present in these preparations must be assembled onto gH/gL, because the purification involved a tag on gH. There were bands of smaller molecular weight proteins in the preparation of trimer containing gO-417, which may be fragments of gO. We quantified the full length gO-417 present in these trimer preparations and observed that gO was approximately 50% that of wild type gO, demonstrating that about half the gH/gL was assembled with gO. Thus, in subsequent experiments, testing the binding of trimers to cells and inhibition of virus entry, we used the gO-417 trimer preparations at twice the protein concentration, compared with wild type gO or gO-251 trimers. Related to this issue with gO-417, experiments detailed below demonstrated that gO-417 trimer bound to PDGFR and to both epithelial and endothelial cells, properties that gH/gL does not possess [33].

We characterized whether trimers containing gO-251 and gO-417 would block HCMV entry into cells, as was the case with trimer containing wild type gO [33]. Fibroblasts or ARPE-19 epithelial cells were incubated with soluble trimers containing w.t gO, gO-251 or gO417 then subsequently infected with HCMV BAD*r*UL131 that expresses GFP in the presence of soluble trimers. Soluble trimer containing wild type gO effectively blocked virus entry into both fibroblasts and epithelial cells (Fig 3B and 3C). By contrast, epithelial cells and fibroblasts treated with trimers containing gO-251 or gO-417 were infected relatively efficiently by HCMV.

### Mutants gO-251 and gO-417 can bind to cell surfaces and interact with PDGFRα

**Fig 4:**
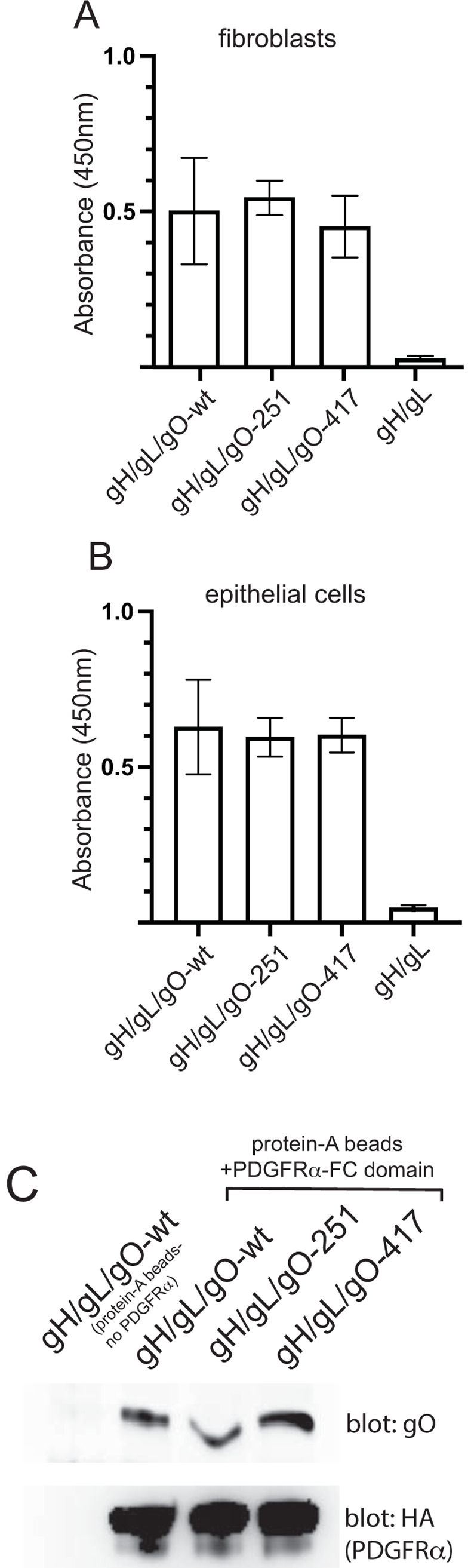
Binding of mutant trimers to cell surfaces and PDGFRα. Fibroblasts (A) or ARPE-19 epithelial cells (B) were fixed with 4% paraformaldehyde in PBS and then incubated with purified soluble trimers containing gO-wt (10 μg/ml), gO-251(10 μg/ml) or gO-417 (20 μg/ml), or gH/gL (without gO) (10 μg/ml) in PBS for 1 h at room temperature. The wells were washed and then incubated with anti-gH MAb AP-86 (4 μg/ml) for 1 h at room temperature then washed and incubated with goat anti-mouse-HRP secondary for 1 h at room temperature. The wells were washed with BPS and incubated with Turbo-TMB colorometric substate and analyzed using a plate reader. Absorbance values are shown on the Y-axis. (C) Soluble PDGFRα-FC-HA was coupled to protein-A agarose then 50μl of beads was incubated with purified soluble trimers containing gO-wt (1 μg), gO-251(1 μg) or gO-417 (2 μg) in 1 ml of Tris-saline for 1h at room temperature. The beads were then washed several times with Tris-saline-0.5% NP-40. Proteins were eluted and separated by SDS-PAGE followed by transfer to PVDF membranes. The membranes were probed with anti-gO rabbit serum or anti-HA Mab (Invitrogen) followed by goat anti-rabbit or goat anti-mouse HRP antibodies. As a negative control, wild-type trimer (1μg) was incubated with protein A-agarose beads without PDGFRα.

To determine whether the trimers containing mutant gO-251 and gO-417 gO could bind to cell surfaces, we incubated the proteins with fibroblasts or epithelial cells then performed cell-based ELISA assays. As previously described [33], the trimer containing gO-wt bound to the surfaces of both fibroblasts and epithelial cells (Fig 4A and 4B). Similarly, trimers containing gO-251 or gO-417 also bound to fibroblasts and epithelial cells with no apparent reduction when compared to trimers containing gO-wt (Fig 4A and 4B).

PDGFRα represents a well-characterized trimer receptor for entry into fibroblasts. We tested whether the mutant trimers could bind to a previously constructed soluble form of PDGFRα, fused to an IgG Fc domain and a HA epitope [52]. This protein was incubated with gO-wt or mutant trimers and PDGFRα-Fc-HA captured using protein-A agarose. Western blot analysis was performed with specific antibodies to detect gO in the trimers that bound to PDGFRα or to PDGFRα itself. The results showed that both gO-251 and gO-417 mutants were able to interact with PDGFRα (Fig 4C). Together, these results show that the mutant trimers bind to PDGFRα, which is essential for virus entry into fibroblasts, and to epithelial cells receptors. The binding of trimers to PDGFRα and cells, along with the assembly of the mutant gO proteins onto gH/gL argue against the notion that these mutant gO proteins are misfolded or globally damaged.

### gO peptides bind Abs present in human sera

**Fig 5:**
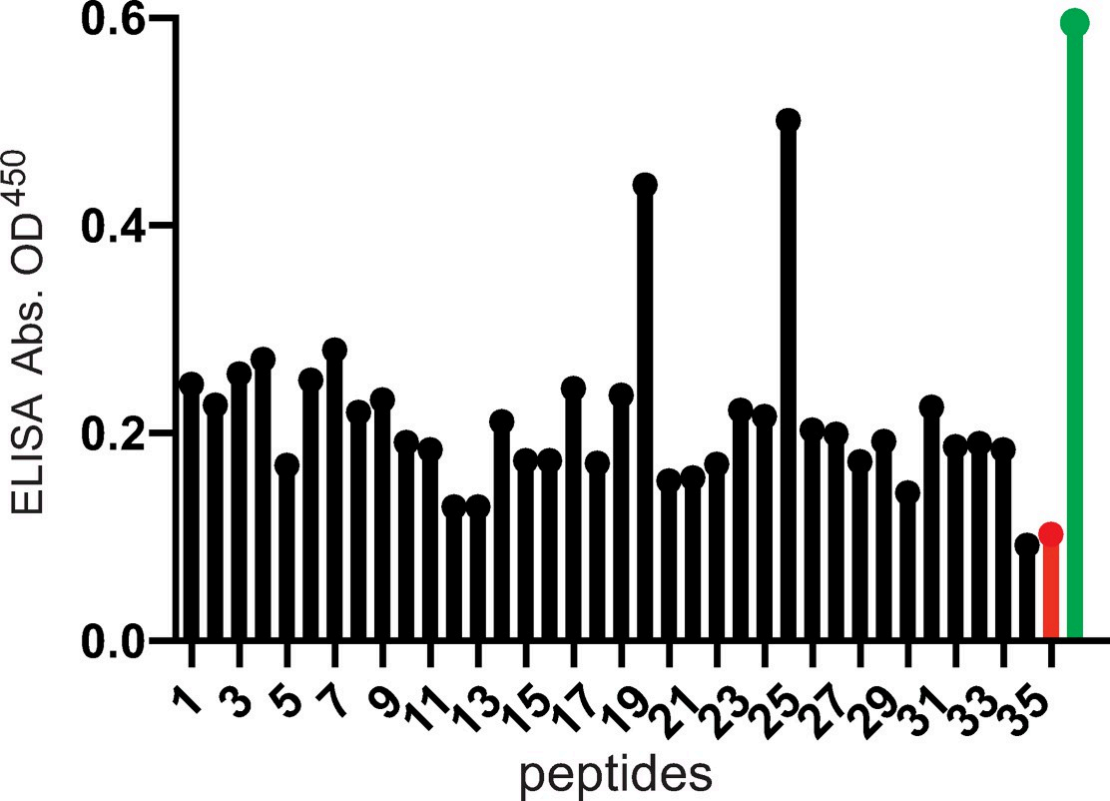
gO peptides recognized by Abs present in HCMV seropositive human serum. The panel of gO peptides with C-terminal biotin tags were incubated in the wells of streptavidin-coated ELISA plates. The wells were washed and then incubated with a pool of 5 different human sera from transplant patients that had been diluted to the NT_100_ titers. The plates were washed and incubated with goat-anti human-IgG-HRP for 1 h at room temperature, washed, and incubated with Turbo-TMB substrate. Control conditions included no peptide plus human sera (red bar) and wells coated with soluble trimer (instead of peptides) and human sera (green bar). The absorbance values are indicated on the y-axis and the numbers of the individual gO peptides are indicated on the x-axis.

The epitopes of NAbs frequently define functionally important domains of viral entry proteins. We showed that similar titers of both pentamer- and trimer-specific NAbs are present in human sera [48]. However, there have been very few reports of gO specific monoclonal antibodies (MAbs) [53] and none that have mapped epitopes in gO. To that end we screened our gO peptide library to determine whether any could bind IgG from human transplant sera, which were previously described by us [48]. The biotin-conjugated peptides were bound to streptavidin coated plates and then incubated with a pool of 5 different human transplant sera that was diluted to a titer which neutralized 100% of HCMV (NT_100_) as described [48]. Peptide 20 (a.a. 248–267) and peptide 26 (a.a. 326–345) stood out as binding higher quantities of IgG compared with other peptides suggesting that these peptides were recognized by substantial quantities of human Abs in the sera (Fig 5). It is important to note that this screen of the peptides did not exclude the possibility that other peptides also bind NAbs.

### Peptides 19/20 and 26 produce NAbs in rabbits

**Fig 6:**
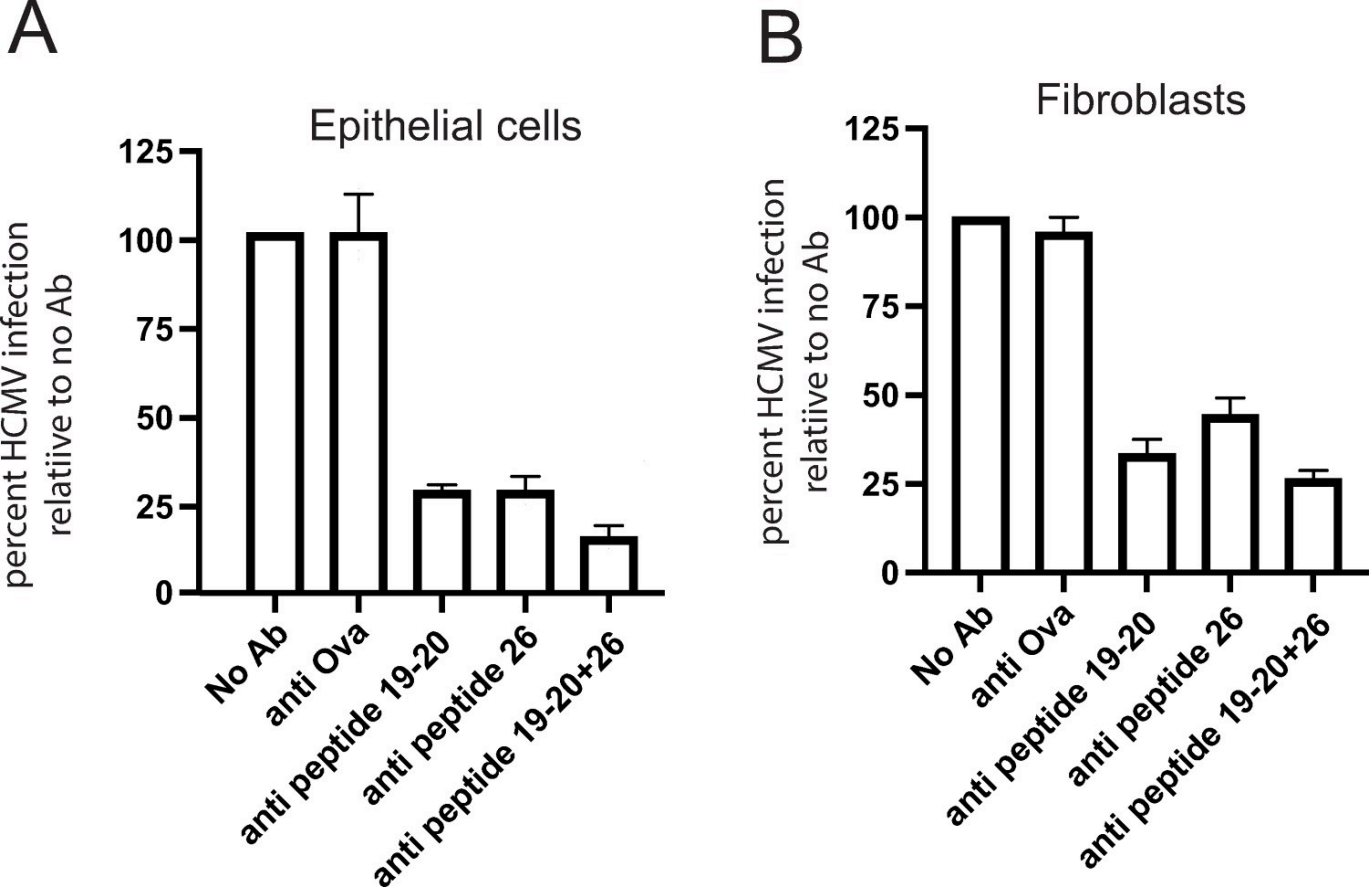
Rabbit polyclonal Abs specific for peptides 20 and 26 neutralize HCMV. (A and B) Rabbits were immunized with peptides 19/20 or 26 conjugated to ovalbumin and peptide-specific antibodies purified using immobilized peptides. Ova-specific antibodies were purified from peptide 19–20 sera using immobilized ova. These Abs were incubated with HCMV BADrUL131 for 1 h at 37°C at 20 μg/ml or 10 μg/ml for fibroblasts or ARPE-19, respectively, The virus and antibodies were then added to cells and incubated for an additional 2 h at 37°C The cells were then washed once with Opti-MEM without FBS and then normal growth media containing Abs was added and the cells and then incubated for 24 hours at 37°C. Virus entry was quantified by counting GFP positive cells from three independent wells and the data expressed as a percentage relative to no Ab control conditions.

To determine whether the sequences within peptides 19/20 and 26 could generate NAbs, a peptide containing all of peptides 19 and 20 (peptide19/20) and a second peptide 26 were conjugated onto ovalbumin (ova) and used to immunize rabbits. Rabbit sera were obtained after 3 rounds of immunization. Peptide-specific Abs were purified using ova-conjugated peptides 19/20 or 26 that had been bound to agarose. As a negative control, Abs in the sera that recognized ovalbumin were purified by incubating sera with ovalbumin bound onto agarose. The purified Abs were eluted, quantified and characterized in virus neutralization assays. When anti-peptide 19/20- or anti-peptide 26 Abs were incubated with HCMV BAD*r*UL131, infection of epithelial cells was reduced by ~75% (Fig 6A). Combining the anti-peptide 19/20 and 26 Abs increased virus neutralization to ~87%. Ova-specific Abs did not reduce HCMV entry (Fig 6A). Similar results were obtained when virus was mixed with these Abs and then used to infect fibroblasts (Fig 6B). However, it is more difficult to neutralize HCMV infection of fibroblasts as entry into cells is more rapid. We concluded that the amino acid sequences in peptides 19/20 (a.a. 235–267) and 26 (a.a. 326–345) were able to induce gO-specific NAbs. In the original screen of these peptides (Fig 5), we did not observe higher levels of Abs that bound to peptide 19 and, thus, most of the NAbs produced in these rabbits likely involved peptide 20.

### Peptides 20 and 26 deplete neutralizing Abs from human sera

To determine whether there were NAbs in human sera that recognize peptides 20 and 26 Abs, we performed Ab depletion experiments, as described [48]. Before these data are presented it is important to understand that 1 μg of either soluble pentamer or soluble trimer was able to substantially reduce virus neutralization by human polyclonal sera [48]. Pentamer and trimer each comprise a substantial fraction of NAb epitopes in any human sera and after serum dilution to NT_100_ titers and depletion with either protein in relative excess reduced neutralization to less than 20%, due in part to the fact that pentamer- and trimer-specific NAbs synergize [48].

**Fig 7:**
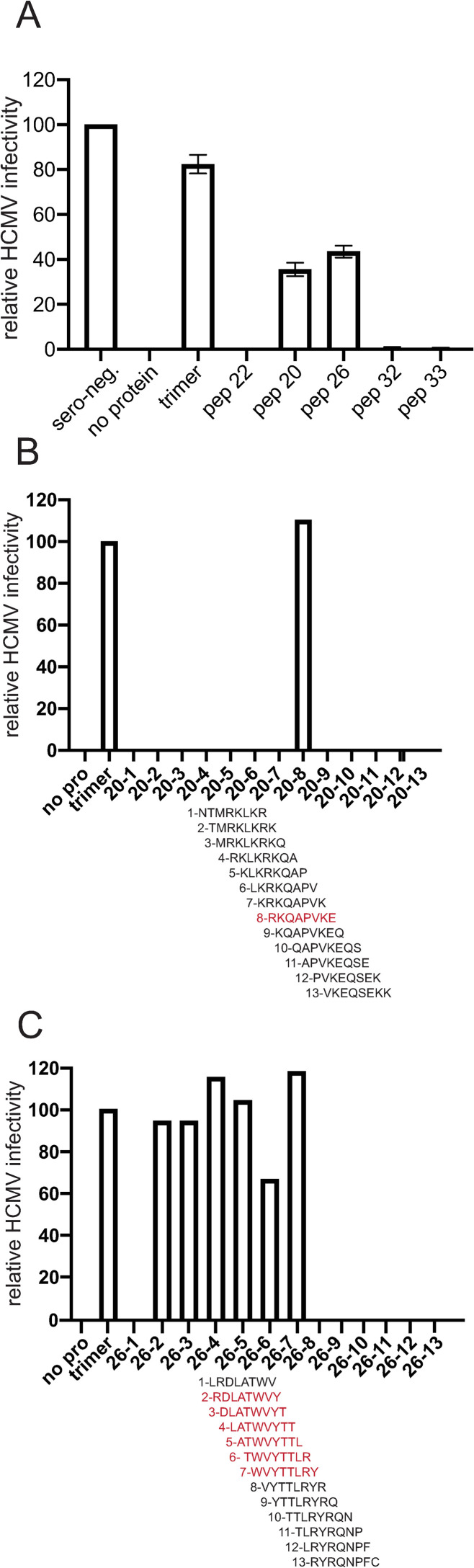
gO peptides 20 and 26 deplete neutralizing Abs from human serum. (A) Pooled HCMV seropositive serum was diluted to (NT_100_) titers then incubated with either no protein, 1μg of soluble trimer, or gO peptides (with biotin): 20, 26, 32 or 33 (each at 0.5 mM) for 1 h at 37°C. Peptide 22, which did not bind substantial IgG, served as a negative control. The trimer was removed using nickel-agarose and peptides removed using streptavidin-agarose. The remaining Abs were tested in neutralization assays involving HCMV (BAD*r*UL131) and ARPE-19 epithelial cells. The relative infectivity of HCMV after incubation with these sera was determined by counting GFP+ (infected) cells after 24 h of infection and compared to the numbers of infected cells following incubation of HCMV with similarly diluted sera from HCMV seronegative donors (sero-neg) Data was collected from three separate wells for each condition. (B and C) Two panels of 8 a.a. peptides with 7 a.a. overlap were derived from peptide 20 (B) or peptide 26 (C). These peptides were tested in depletion assays as in panel A. The relative infectivity of HCMV (BAD*r*UL131) on ARPE-19 epithelial cells was determined and normalized to the level of HCMV infectivity with depletions performed with soluble trimer. Data was collected from a randomly selected field of the same surface area from each well.

We previously determined the NT_100_ values for several human sera using a serial dilution assay as previously described [48]. For these experiments, a pool of 5 human transplant sera diluted to NT_100_, was incubated with 1 μg of purified soluble trimer or peptide 20 or 26 (0.5mM) conjugated to biotin. The peptides or trimer were removed using streptavidin (peptides) or nickel-agarose (trimer). The depleted sera were then tested in neutralization assays involving the GFP-expressing HCMV BAD*r*UL131 as described [48]. Pooled seropositive human sera not treated with trimer or peptides (no protein) completely neutralized HCMV infectivity (Fig 7A). By contrast, sera incubated with soluble trimer exhibited 82% infectivity and sera incubated with peptides 20 and 26 displayed 37% and 45% infectivity, respectively (Fig 7A). As a control, sera that was incubated with peptide 22, which did not bind substantial IgG from human sera (Fig 5), retained complete neutralizing capacity of HCMV. Interestingly, incubation of sera with peptides 32/33, which could block HCMV entry also did not deplete NAbs. We concluded that, epitopes in peptides 20 (a.a. 248–267) and 26 (a.a. 326–345) are recognized by naturally induced human NAbs.

Observations of substantial loss of NAb activities after incubation with trimer (82% in Fig 7A) or peptides (85% with peptide 20–8 Fig 7B) might be difficult to comprehend because there are reports of the importance of pentamer-, gH/gL- and gB-specific NAbs. Pentamer and trimer were able to substantially deplete NAbs in a large panel of human sera we tested before [48]. Another group observed that pentamer could substantially (76%) deplete NAbs in human sera [45]. It is important to recognize that these sera were diluted to NT_100_ titers (1:320) before incubation with pentamer, trimer or peptides in large excess and then neutralization was frequently reduced by over 60% and, with some sera, by 90–100%. Our results in no way suggest that there are not NAbs specific for gB or gM/gN or other HCMV proteins. Indeed, it would be very surprising that Abs specific to gB (especially the pre-fusion form) were not neutralizing. Virus neutralization with polyclonal sera is a highly synergistic process. One way of thinking about this is to picture an army made up of different forces: artillery, foot soldiers and cavalry. It you remove the artillery entirely from the fight, you might lose the battle despite the cavalry and foot soldiers. We did not compare our peptides one to another or to full-length trimer in dilutions experiments to determine the relative effects of peptides or trimer for two reasons; i) our aim was to define NAb epitopes in gO not quantify their relative effects and ii) synthetic peptides are unlikely to be as potent as folded proteins. All that said, our results clearly show that there are NAbs that recognize gO sequences represented in peptides 20 and 26.

We refined the mapping of NAb epitopes within peptides 20 and 26 using panels of 8 a.a. peptides overlapping by 7 a.a. (S2 Table) to identify smaller peptides that could deplete neutralizing Abs from human sera. Only one peptide derived from peptide 20, peptide 20–8 R_255_-E_262_, substantially depleted Nabs from the sera (Fig 7B). Several peptides in the N-terminal half of peptide-26 depleted NAbs from the human sera identifying sequences R_327_-Y_339_ as containing important gO epitopes (Fig 7C).

### Peptides 20 and 26 can purify NAbs from human sera

**Fig 8:**
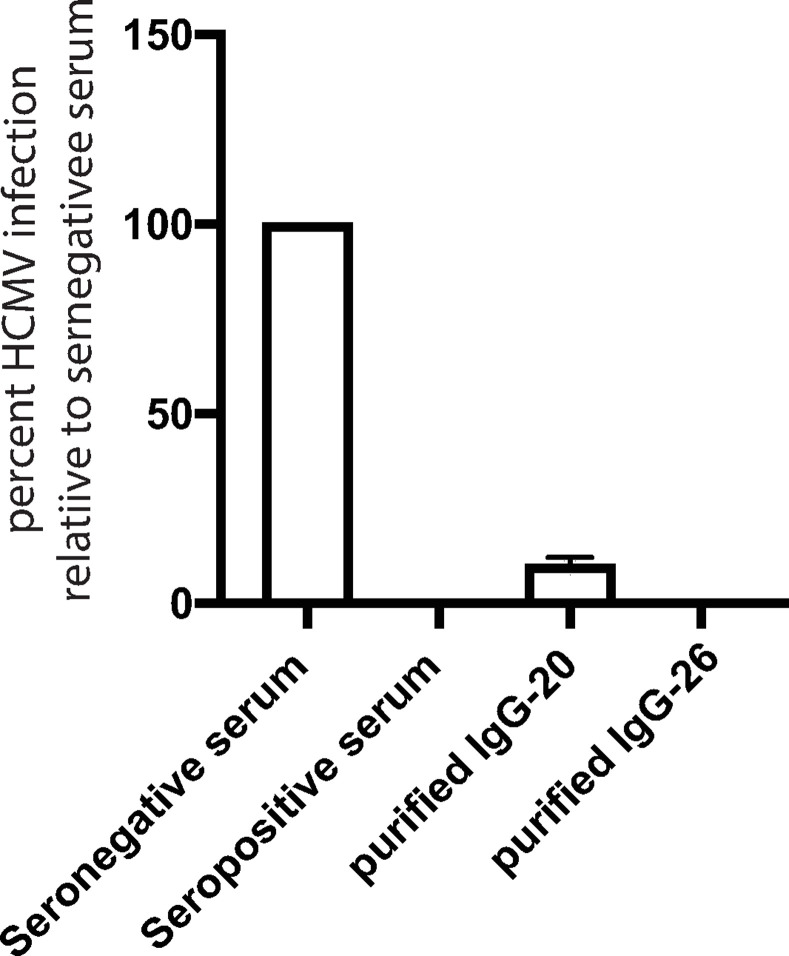
gO peptides pull out neutralizing Abs from human serum. Biotinylated peptides 20 or 26 were covalently immobilized onto agarose (AminoLink) and incubated with pooled human transplant sera for 1 h. The agarose beads were washed with AminoLink wash solution then Abs bound to the peptides were eluted in 0.1 M glycine-pH 2.2, quickly neutralized and buffer exchanged using Zeba columns (ThermoFisher). HCMV BADrUL131 was incubated for 1 h at 23°C with either pooled seronegative or seropositive human sera (1:320 dilution, NT_100_ titer) or with peptide-purified anti-peptide 20 or 26 Abs at 1μg/ml IgG. The virus and sera or Abs were applied to ARPE-19 epithelial cell monolayers and incubated for 2 h at 37° C, then the cells washed and replenished with growth media containing sera or IgG then incubated 24 h at 37° C. Virus infectivity was assessed by monitoring GFP expression, comparing the number of GFP positive cells to that observed when HCMV was incubated with seronegative sera.

To extend these observations, we tested whether we could pull NAbs from human sera using these peptides. Pooled human transplant sera were incubated with peptides 20 and 26 covalently bound to agarose. The Abs that bound were eluted in low pH buffer, quickly neutralized, then buffer exchanged. These Abs were then tested for their capacity to neutralize HCMV. As controls, sero-negative sera did not neutralize HCMV while pooled seropositive sera (at the NT_100_ dilution) effectively neutralized HCMV (Fig 8). Abs that bound to peptide-20 (1 μg/ml) neutralized ~90% of the HCMV and Abs that bound to peptide 26 (1 μg/ml) neutralized 100% of the HCMV infectivity. To summarize the second part of the paper, two important gO NAb epitopes were identified. One involved peptide 20 including the sequence R_255_-E_262_ and peptide 26 containing a sequence R_327_-Y_339_. Peptides 32/33 including the overlapping sequences D_417_YLDSLL were important for virus entry, but did not obviously bind NAbs.

## Discussion

To identify functionally important sequences or domains in gO that promote virus entry, we characterized an overlapping panel of 20 a.a. peptides for inhibition of HCMV entry. Obviously, this approach has a significant disadvantage in that the peptides can only represent domains in gO that can be formed by 20 a.a. linear sequences. Any gO domains formed by folding of sequences that are more distant from one another would not be represented. In addition, our peptide screen could potentially not identify important domain due to sequences differences in the N-terminal region of gO polypeptides from different stains (e.g. TR versus AD169). However, this approach did identify two sets of overlapping peptides: i) peptides 19 and 20 that together span a total of a.a. 235–267 and ii) peptides 32 and 33 that span a.a. 404–436. All four of the peptides blocked virus entry into both fibroblasts and epithelial cells. Thus, the 7 a.a. sequences in these pairs of peptides that are shared: N_248_TMRKLK in peptides 19/20 and D_417_YLDSLL in peptides 32/33 are important, and likely sufficient, for this inhibition. Note that both the 19/20 and 32/33 sequences are highly conserved in all sequenced gO proteins [49]

To extend these results to mutations in the whole trimer molecule, we mutated sequences within the 7 a.a. peptide 19/20 and the 32/33 gO consensus sequences, producing: gO-251 and gO-417. The mutant gO proteins were coexpressed along with a soluble form gH (with the transmembrane domain removed) and gL in 293-6E cells and soluble trimers were purified from culture supernatants. Both gO-251 and gO-417 assembled to produce trimers that were secreted from cells. Mutant trimers containing gO_251_ or gO_417_ did not block HCMV entry into either fibroblasts or epithelial cells, whereas trimer with gO-wt blocked entry. However, trimers containing either gO-251 or gO-417 bound to PDGFRα and to fibroblasts and epithelial cells. This was surprising and is discussed below. We concluded peptide 19/20 and 32/33 sequences represent two functionally important domains of gO.

The 19/20 peptides contained the 7 a.a. consensus sequence: N_248_TMRKLK that are present in a highly conserved region of the gO polypeptide extending from a.a. 202 to 267, comparing 40 HCMV clinical strains and several lab strains [49]. The gO sequences present in this region are the largest and most conserved region of gO, yet gO is among the least conserved of HCMV proteins [54,55]. The AD169 gO described in the Rasmussen paper, contains an identical sequence N_256_TMRKLKRKQ (that is moved to the right compared to HCMV strain TR by insertion of more N-terminal sequences). Stegmann and colleagues [39] mutated 13 conserved domains of gO, in this case comparing gO sequences from monkey and mouse and other species of cytomegalovirus [39]. They then transferred mutations into the HCMV gO gene in the virus genome. Most of their mutations had no negative effects or abolished trimer assembly, but one mutant HCMV expressing gO R_249_KLKRK_254_ ➔ AALAAA (this sequence is identical to our R_251_KLKRK but is shifted in their HCMV strain TB40 gO) displayed reduced entry of cell free virus. Thus, the results of Stegmann and colleagues report [39] fit well with our results, testifying to a fundamentally important domain gO in the region R249-254, which encompasses a conserved, highly charged sequence.

The second functionally important domain in gO was identified using peptides 32/33, which inhibited HCMV entry as effectively as peptide 20 and better than peptide 19 at lower concentrations (7.8 uM). These peptides contained the consensus sequence D_417_YLDSLL_423,_ which was present within a larger sequence containing numerous aspartic acid, tryptophan, serine and threonine residues. As with peptides 19/20, peptides 32/33 are well conserved in other HCMV gO molecules, but peptide 32/33 sequences were not mutated by Stegmann and colleagues [39]. The mutant trimer containing gO-417 with only two substitutions: D_417_YLD ➔ AYLA was unable to block entry of HCMV. One might argue that gO-251 or gO-417 were globally misfolded proteins. However, this suggestion is not consistent with our observations that both proteins assembled with g/gL and trimers containing these mutant gO proteins could bind to PDGFRα and to epithelial and fibroblast cells.

**Fig 9:**
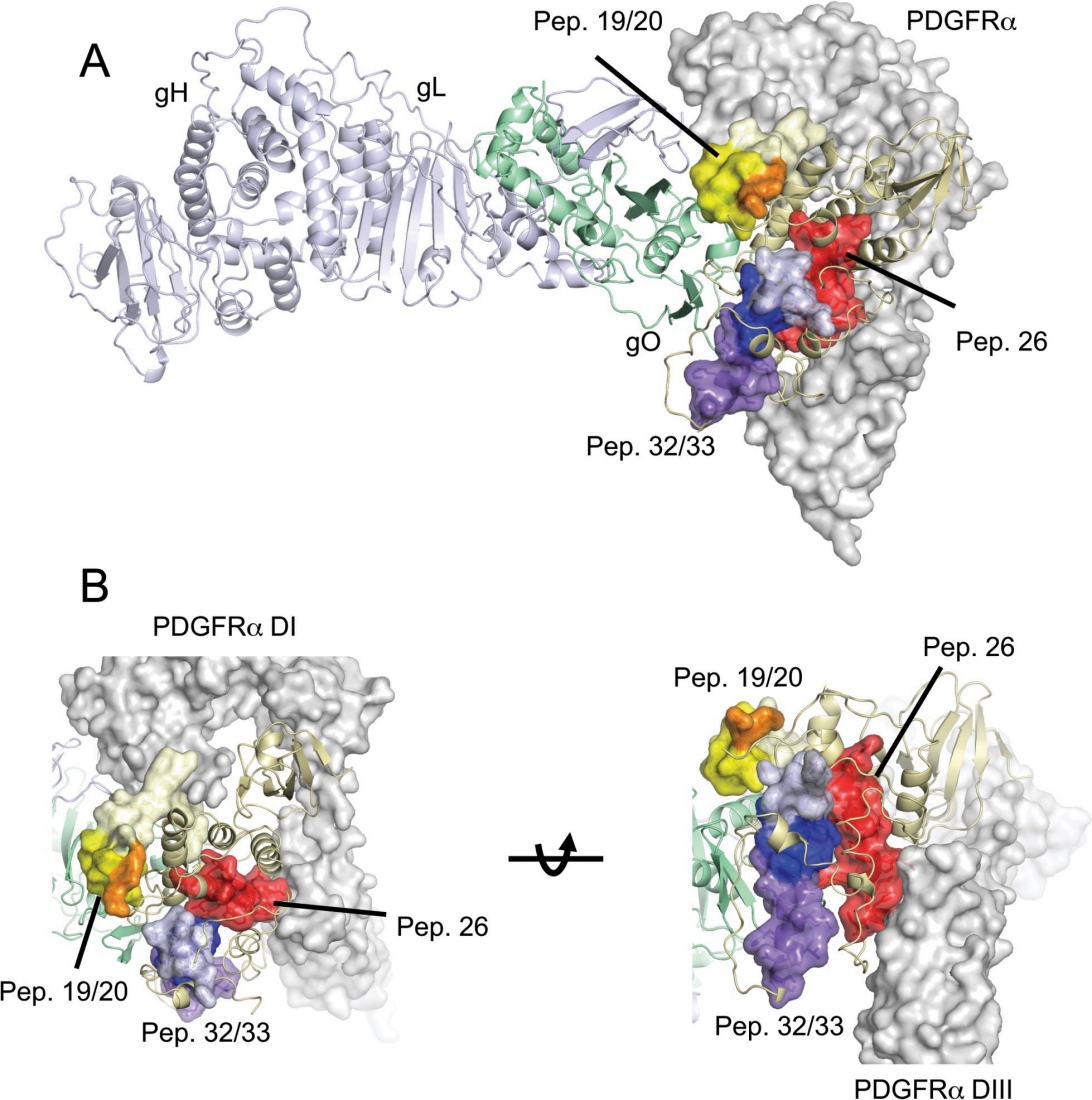
Localization of gO peptides in the structure of trimer bound to PDGFRα. (A) Peptides 19/20, 26 and 32/33 are shown as colored surfaces on the structure of gHgL^AD169^gO^TR^:PDGFRα [40]. Pep19/20 is colored yellow and orange, with the unique residues from pep19 (residues 235–247) colored light yellow, the overlapping residues in peptides 19 and 20 were colored bright yellow (residues 248–254) and the unique pep20 residues colored orange. Note that the C-terminal portion of pep20 (residues 256–267) is in a flexible loop that is not visible in the cryo-EM structure. Pep32/33 is colored blue and purple. The unique residues of pep32 (residues 404–416) are colored light blue, the overlapping residues in pep32 and pep33 are colored dark blue and the residues unique to pep33 are colored purple. Pep26 is colored red. The gH/gL/gO trimer is shown in cartoon format, with gH colored light blue, gL colored light green and gO colored light yellow. PDGFRα is shown as a semitransparent surface colored grey. Panel B shows two different closeup orientations of the gO peptides relative to PDGFRα. The N-terminal region of pep19 makes contacts with PDGFRα DI.

Fig 9 shows the positions of peptides 19, 20, 32 and 33 in our recently acquired structure of the HCMV trimer (AD169 gH/gL with TR gO) bound to PDGFRα [40]. gO forms a globular structure attached to gL that interacts with three N-terminal domains of PDGFRα (DI-DIII), similar to the structure described by Kschonsak et al. [36]. In Fig 9, unique sequences in peptide 19 were colored light yellow, unique sequences in peptide 20 in orange and the 19/20 overlapping sequences colored bright yellow. The 19/20 overlapping sequences (bright yellow) form a prominent lobe extending from the surface of gO and away from PDGFRα (Fig 9). Note that the N-terminus of peptide 19 is oriented toward the DI region of PDGFRα, but this sequence is not found in peptide 20 and, thus, not necessary for function. No residues of peptide 20 including the consensus sequences are in contact with PDGFRα, which is on an opposite facing surface of gO. Peptides 32 and 33 together form an elongated structure on the surface of gO with two lobes, one composed of unique peptide 32 sequences (light blue) and the other of unique 33 sequences (purple), both pointing away from PDGFRα. The overlapping sequences of peptides 32 and 33 (dark blue-purple) form a concave structure between these two lobes (Fig 9A). This 32/33 concave surface is in a similar orientation and adjacent to the peptide 19/20 overlap surface. This modeling of the peptides on the structures of trimer and PDGFRα fits well with our observations that mutations in peptide 19/20 and 32/33 consensus sequences do not alter binding to PDGFRα or, apparently, to epithelial cell trimer receptors. Thus, the protein sequences included in peptides 19/20 (including the mutations in gO-251) and 32/33 (gO-417) are present on the opposite face of the trimer from the surface of gO that interacts with PDGFRα. Given that gO contacts other proteins (TGFbRIII) via the PDGFRα binding surface, it is perhaps likely that trimer also binds other biologically relevant receptors (on epithelial and endothelial cells) by this PDGFRα -binding surface.

Our previous observations that soluble trimer blocked HCMV entry into fibroblasts and epithelial cells suggested that soluble trimer molecules compete with HCMV for cell surface receptors, receptors that are essential for virus entry [33]. The affinity of soluble trimer for PDGFRα is high (2 X 10^−9^) [28] and inhibition of entry into fibroblasts was 90% with 1.5 μg/ml of soluble trimer. However, here, mutant trimers with gO-251 (peptide 19/20) or gO-417 (peptide 32/33) could bind to epithelial cells and fibroblasts and to PDGFRα and yet could not block virus entry. These results suggest that trimer inhibition of HCMV entry is not explained entirely by competition of soluble trimers for receptors, but involves other effects of trimer that are important for virus entry. Given all we know from how herpesviruses enter cells, it seems most likely, but not proven, that these effects of trimer represent steps in virus entry that occur after receptor binding, especially because the mutant soluble trimers can bind to cells and PDGFRα. Normally, receptor binding precedes other steps in virus entry leading to membrane fusion in the case of HCMV in endosomes.

To attempt to explain these surprising results we can conceive of three models for how the 19/20 and 23/33 sequences (mutated in soluble trimers gO-251 and gO-417) could function to promote HCMV entry into different cell types: epithelial cells and fibroblasts. The first model suggests that the gO-251 and gO-417 mutations eliminate binding to important receptors that are essential for HCMV entry, but do not abolish binding to cell surface proteins that are irrelevant for entry (so the mutant proteins continue to bind to cells). There are several reasons to dismiss this model. First, for fibroblasts, PDGFRα is necessary and sufficient (as a receptor) for entry into fibroblasts and the gO-251 and gO-417 mutant trimers bind to PDGFRα, but do not block entry. Thus, soluble trimer binding to receptors other than PDGFRα in fibroblasts would not explain our observations. One might argue that mutant trimers bind to PDGFRα with lower affinity and, thus, do not compete as well with wild type trimer that is present in the virus. But this suggestion does not fit well with the coimmunoprecipation of PDGFRα and to binding epithelial cells and observations that the gO-251 and gO-417 sequences were distant from PDGFRα binding surfaces of gO (Fig 9). The HCMV trimer receptors present on epithelial cells have not been identified. However, trimer binds to two other molecules that do not mediate HCMV entry. A highly oligomerized form of trimer bound to NRG2, but the affinity was extremely low so that a soluble form of trimer would not bind to NRG2 [35]. NRG2 was not considered relevant in a subsequent paper [36]. Soluble trimer binds with high affinity to TGFbRIII, but silencing of TGFbRIII and soluble forms of TGFbRIII produced no inhibition of HCMV entry (Kschonsak et al. 2021) [36]. Importantly, the gO251 and gO417 sequences are present in a region of gO that is distant from the gO surfaces that bind both PDGFRa and TGFbRIII. Thus, the gO-251 and gO-417 sequences are functionally important–mutations inhibit the capacity of trimers to block virus entry, yet these sequences are apparently distant from recognized trimer receptor binding domains (PDGFRα or important epithelial cell receptors). For these reasons, we do not favor this model. However, the identification of the relevant epithelial receptors and efforts to determine if trimers can bind to other cell surface proteins will be necessary to test this model.

The second model to explain our results suggests that the sequences represented in gO peptides 19/20 and 32/33 are involved in trimer interactions with gB that trigger entry fusion. Once HCMV arrives in endosomes of fibroblasts or epithelial cells [31,56], gB must be triggered for entry fusion, delivering HCMV capsids into the cytoplasm. In epithelial and endothelial cells, pentamer interacts with NRP-2 for virus entry [35] so that gB might be triggered by pentamer for entry fusion. However, in fibroblasts, pentamer is not necessary for entry, which strongly suggests that trimer interacts directly with gB to promote entry fusion. Thus, soluble forms of trimer with the gO-251 and gO-417 mutations might be unable to inhibit these trimer-gB interactions, something that w.t. trimer can do. Related to this hypothesis, the surfaces of the 19/20 and 32/33 peptides represent gO sequences that reside side-by-side on a surface of gO pointing away from the PDGFRα-binding surfaces, which include a lobe from peptides19/20 and a depression formed by peptides 32/33. These sequences might fit onto a surface of gB. This model is our favored one because triggering of gB for entry fusion is a process that trimer must be involved in, yet it makes ample sense that the gB-binding surface on trimer is distant from the receptor binding surface.

The third model for how the gO-251 and gO-417 surfaces might function during virus entry, relates to observations that trimer binding to cell surface receptors is followed by traffic of virus in the plasma membrane to sites of macropinocytosis followed by incorporation into endosomes [31,56]. In this model, trimer may interact with cellular or viral proteins that increase cell surface traffic or macropinocytosis or egress from endosomes. By this model, our peptides and w.t. soluble trimer might disrupt interactions with these viral or cellular proteins so that virus particles do not reach the cytoplasm. One example of such a protein is the HCMV pentamer that is not required for virus binding to epithelial cells or for macropinocytosis but is required for release from endosomes [31,33]. Cell trafficking proteins might also be involved in entry. Whatever the mechanism of action of these gO sequences, our studies highlight functionally important surfaces on gO, which are distinct from known receptor binding surfaces and that appear to act downstream of receptor binding.

The second half of the paper focused on defining epitopes recognized by NAbs in pools of human transplant sera. This provided a different approach to defining functionally important domains of gO. Again, the caveat here is that the peptides can only identify epitopes formed by continuous amino acid sequences. That said, this approach highlighted two functionally important regions of gO. Peptides 20 and 26 bound relatively higher quantities of IgG from the human sera compared with other peptides. These peptides produced rabbit polyclonal Abs that were neutralizing and the peptide 20- and 26-specific Abs produced additive effects. These two peptides were also able to pull out NAbs from human transplant sera. Interestingly, peptides 32 and 33 that blocked HCMV entry were not obviously recognized by NAbs in these sera. The NAb epitopes in peptide 20 were able to recognize an 8-mer in the center of peptide 20: R_255_KQAPVE. This sequence was directly adjacent to N_248_TMRKLK 19/20 peptide consensus sequence, providing further evidence that this highly charged lobe that protrudes from the surface of gO is important for HCMV entry.

NAbs also recognized peptide 26 describing a third functionally important epitope in gO. The NAb epitopes in peptide 26 extended across the N-terminal half of the peptide including a wider footprint R_327_DLATWVYTTLRY. Peptide 26 differed from peptides 19/20 and 32/33 in that most of peptide 26 was in direct contact with PDGFRα domain III (Fig 9). Thus, peptide 26-specific Abs might block receptor binding and this is being tested both by producing monoclonal Abs and by mutating the protein sequences. The affinity of trimer for PDGFRα is very high, there are three domains of gO that contact PDGFRα, and mutating individual contact residues often does not inhibit binding [36]. However, an anti-peptide 26 antibody would likely have a large footprint in order to block receptor binding.

In summary, our studies identified three functionally important domains in gO. Two of these domains represented by the consensus sequences of peptides 19/20 and 32/33 formed a lobe (19/20) and depression (32/33) on a surface of gO that points away from the PDGFRα-binding domain. Thus, our results indicate these combined surfaces of gO represent a functionally important protein surface that is conserved in gO proteins. This provides the first evidence that the HCMV trimer is involved in a novel post-receptor binding mechanism that promotes virus entry. In our screen for peptides recognized by NAbs, peptide 20 was again prominent, suggesting that NAbs recognize the 19/20 lobe and can block this post-binding entry step. Peptide 26 was also recognized by NAbs and these sequences contact PDGFR, so that peptide 26-specific Abs may inhibit the receptor binding step in entry. The peptide 19/20 lobe and peptide 26 sequences represent the first described NAb epitopes in gO.

## Material and methods

### Cells and viruses

Primary human neonatal dermal fibroblasts (NHDFs) were obtained from ATCC and grown in DMEM with 10% (fetal bovine serum0 FBS. Human retinal pigmented epithelial (ARPE-19) cells were obtained from ATCC and grown in DMEM/F12 plus 10% FBS. All cells were maintained at 37°C with 5% CO2. The HCMV BAD*r*UL131 (kindly provided by Tom Shenk, Princeton University) is a derivative of AD169 that has had the UL131 gene repaired to allow for expression of pentamer and encodes a GFP reporter gene. HCMV stocks were produced from NHDFs grown in roller bottles and viral particles were concentrated from culture supernatants by centrifugation through a cushion of 20% sorbitol in PBS at 80,000 × g for 1 h. Pellets were suspended in DMEM plus 10% FBS and frozen at -70°C. HCMV stocks were tittered by determining the number of infectious units per ml (IUs ml^-1^) by serial diluting virus stocks and then adding the dilutions onto NHDF monolayers and staining for the HCMV immediate early gene IE-86 after 24 with anti-IE-86 rabbit polyclonal serum 6658. HSV-VP26-GFP that has the GFP marker fused to the VP26 tegument protein has been described previously [57].

### Virus entry and neutralization assays

HCMV entry assays were performed with BAD*r*UL131 supernatant-derived virus using a dose of either 0.5 or 5 infectious units (IU) per cell for fibroblasts or epithelial cells, respectively. For entry assays involving peptide inhibition, cell monolayers were incubated with peptides for at 1 h at 4°C and then HCMV BAD*r*UL131 virus was added to the cell monolayers and incubated for 1 h at 4°C. The cells were then shifted to 37°C and allowed to incubate for another 2 h. The cells were then washed once with Opti-MEM without FBS and then normal growth media containing peptides was added and the cells incubated for 24 hours at 37°C. For entry assays involving soluble proteins, cells monolayers were incubated with soluble protein complexes for 1 h at 4°C as previously described [48] and then incubated at 4°C for an additional 1 h after adding virus. The cells were then shifted to 37°C and allowed to incubate for another 2 h. The cells were then washed once with Opti-MEM without FBS and then normal growth media and then incubated for 24 hours at 37°C. For Ab or sera neutralization assays, HCMV BAD*r*UL131 was incubated with Abs or heat inactivated sera for 1 h at 37°C and then added to cell monolayers for an additional 2 h then the cells washed once with Opti-MEM without FBS and then normal growth media containing sera or Abs was added and the cells incubated for 24 hours at 37°C. Virus entry was measured by assessing GFP expression by fluorescent microscopy.

### Soluble proteins

Construction, expression and purification of wild type soluble trimer was described [33]. For construction of mutant forms of gO the following oligonucleotides were used:5’-AAGAACACCATGGCAGCACTAGCCGCCGCGCAGGCCCCCG-3’, 5’-CGGGGGCCTGCGCGGCGGCTAGTGCTGCCATGGTGTTCTT3’-were used to substitute amino acids 251-RKLKRK-256 to 251-AALAAA-256 and oligonucleotides 5’-ACCCCCTGTGGGCATACTTAGCCAGCCTGCTGTTCCT3’,5’-AGGAACAGCAGGCTGGCTAAGTATGCCCACAGGGGGT -3’ were used to make the amino acid substitutions 417-DYLD-420 to 417-AYLA-420. PCR products were generated using the mutating primer in conjunction with oligos 5’-CCTCCCATATGTCCTTCCGAGTG-3’ and 5’-ACACTTGAGTGACAATGACATCC-3’ that are specific to plasmid p-TT5 [58] using a standard overlap-extension PCR protocol. These mutant gO plasmids were cotransfected along with a soluble form of gH and gL into HEK293-EBNA1-6E cells (Canadian NRC) following NRC protocols using linear polyethylenimine 343 (PEI) at a 1:3 plasmid to PEI ratio. Six days after the transfection, the cell culture supernatants were harvested and soluble trimers purified as described [33].

### Serum depletion with Abs

Human sera from heart transplant patients were obtained at Oregon Health & Science University with patient consent (OHSU IRB Protocol #0004474 as described previously [48]. These sera were pooled from 5 or 6 donors then diluted to NT_100_ titers in Opti-MEM without FBS and incubated with 1 μg soluble trimer [48], or 0.5 mM of peptide for 1 h at 37°C. Either 15 μl of nickel NTA agarose (Invitrogen) or streptavidin agarose (EMD Millipore) was added to the samples followed by incubation at room temperature for 1 h with agitation. The sera were then centrifuged through affinity purification spin columns (Pierce) at 1000 × g for 30 sec to separate the sera from agarose. These sera were then tested in HCMV neutralization assays as described above.

### Peptides and antibodies

An overlapping peptide library spanning the entire length HCMV gO polypeptide from strain TR was obtained from Genscript Biotech, Piscataway, NJ. The peptides consisted of 20 amino acids in length with 7 amino acid overlap and a biotin moiety conjugated to the C-terminus. A panel of 8 a.a. peptides overlapping by 7 a.a. and a biotin moiety conjugated to the C-terminus that were derived from the protein sequences: 248-NTMRKLKRKQAPVKEQSEKK-267 or 326-LRDLATWVYTTLRYRQNPFC-345 (S2 Table) were also obtained from Genscript Biotech. Peptides derived from the gO polypeptide sequence 235-FRVPKYINGTKLKNTMRKLKRKQAPVKEQSEKK-267 (peptide 19/20 from overlapping library) and 326-LRDLATWVYTTLRYRQNPFC-345 (peptide 26 from overlapping library) were synthesized conjugated onto ovalbumin (ova). These ova-peptides were used to produce New Zealand White rabbit polyclonal sera by priming rabbits with ova-peptides in Freund’s adjuvant then boosting the rabbits with ova-peptides in TiterMax Gold adjuvant (Sigma, St. Louis, MO). Rabbit antibodies specific for peptide 20, 26 and ovalbumin were purified using peptides ova-20 and ova-26 or ova (without peptides) by coupling these proteins to AminoLink (Thermo Scientific).

### Purification of antibodies from human serum

Ova-conjugated peptides 20, 26 or ovalbumin were immobilized to agarose (Amino-Link, ThermoFisher) and then incubated with pooled human sera that has been previously described [48]. The bound antibodies were eluted in 0.1M glycine-pH 2.2 and quickly neutralized with 1/10^th^ volume of 0.1M Tris-pH 9.0 and then buffer exchanged into Tris-Saline pH-7.2 using Zeba buffer exchange columns (ThermoFisher). Antibodies were quantified by SDS-PAGE followed by coomassie staining.

### ELISA assays

For cell-based ELISA assays, ARPE-19 epithelial or fibroblast cells in 96-well plates were fixed with 4% paraformaldehyde, washed with PBS supplemented with 0.1M glycine pH.7.2, then washed with PBS plus 2% BSA and incubated with soluble trimers or gH/gL complexes at 10 μg/ml (or 20 μg/ml in the case of gO-417) in PBS with 2% BSA at room temperature for 1 h. The cells were then washed with PBS with 2% BSA followed by incubation with anti-gH Mab AP-86 (4 μg/ml) in PBS plus 2% BSA for 1h followed by several washes with PBS with 2% BSA. A goat-anti-mouse-HRP antibody was diluted 1:2,000 in PBS with 2% BSA and added to the wells and incubated at room temperature for 1 h then the cells washed several times with PBS plus 2%BSA. In the screens involving human antibodies binding to peptide, biotin-conjugated gO peptides (0.5 mM) were incubated in streptavidin-coated 96-well plates (Pierce) overnight at 4°C. The wells were then washed with PBS and then incubated with pools of HCMV seropositive human sera diluted in PBS to the NT_100_ titers for 1 h at 23°C. The wells were then washed several times with ELISA wash buffer and then incubated with goat anti-human-IgG antibodies conjugated with horse radish peroxidase diluted to 1:2,000 in ELISA buffer for 1 h at 23°C. In both ELISA assays, 100μl of Turbo-TMB substrate was added to the wells and incubated at room temperature until signal developed. The colorometric reaction was stopped by adding 100 μl of 1M sulfuric acid to the wells and then the substrate development was read in a Molecular Dynamics precision plate reader at a wavelength of 450 nm.

### Immunoprecipitations and western blotting

Soluble PDGFRα fused to an Fc domain and HA epitope tag was expressed in 2936E cells and coupled to protein A agarose as previously described [33]. Purified soluble trimer complexes were added to 1ml of Tris-saline buffer (50 mM Tris-HCl, pH 7.4, 150 mM NaCl) with 50 μl of the PDGFRα-coupled protein-A agarose or protein-a agarose alone and incubated at room temperature while rotating. Protein complexes were collected by centrifugation at 500×g, washed 3 times with Tris-saline (50 mM Tris-HCl, pH 7.4, 150 mM NaCl, 0.5% NP-40) and eluted in sample loading buffer (50mM Tris-pH 6.8, 10% glycerol and 2% SDS) with 1% 2-mercaptoethanol. Precipitated proteins were separated using SDS-polyacrylamide electrophoresis and then transferred to polyvinylidene fluoride (PVDF) membranes. Membranes were incubated in TBS containing 0.1% Tween-20 (TBST) plus 5% non-fat milk, washed, followed by incubation in TBST with antibodies specific for HA (Invitrogen) or a rabbit polyclonal sera specific for gO [59] overnight at 4°C. Membranes were washed 3 times for 10 min in TBST and incubated in TBST with horseradish peroxidase-conjugated secondary antibodies for 1h. Proteins were detected by incubating membranes in chemiluminescent reagent (Perkin Elmer) and imaged with an Imagequant LAS 4000 system (GE Healthcare).

## Supporting information

S1 Fig Peptide inhibition of virus entry. ARPE-19 monolayers were incubated with gO peptides 19, 20, 32, 33 or control peptide 21 (100 μM each) at 4° C for 1 h then HCMV BADrUL131 virus (MOI 5)) was added to the cells and then incubated at 4° C for 1 h. The cells were then shifted to 37° C for 2 h. The cells were then washed and incubated in fresh growth media containing peptides at 37° C for 24 h. The level of virus entry was determined by assessing GFP expression by fluorescent microscopy and comparing to control conditions (peptide 21) and expressed as percent infection relative to the control.
(EPS)

S1 Table Peptide library derived from HCMV gO (strain TR) sequence. Listed are the numbers of the peptide followed by the amino acid sequence and the residue coordinates that define the peptide.
(DOCX)

S2 Table Peptide library derived from the amino acid sequences of peptide 20 or 26 shown in S1 Table. Listed are the numbers of the peptide followed by the amino acid sequence and the residue coordinates that define the peptide.
(DOCX)
