# Supplementary material for: Identification of functionally important domains of human cytomegalovirus gO that act after trimer binding to receptors
Source: PLoS Pathog. 2022 Apr 22;18(4):e1010452. doi: 10.1371/journal.ppat.1010452 (PMC9032346; doi:10.1371/journal.ppat.1010452)
Supplement: S1 Table — Listed are the numbers of the peptide followed by the amino acid sequence and the residue coordinates that define the peptide. (DOCX) [file ppat.1010452.s002.docx]

| gO 1 | 1-MGRKGEMRGVFNLFFLMSLT-20 |
| --- | --- |
| gO 2 | 14-FFLMSLTFLLFSFINCRAAV-33 |
| gO3 | 27-INCRAAVRLSVGRYWSGKVL-46 |
| gO4 | 40-YWSGKVLSTIGKQRLDKFKL-59 |
| gO5 | 53-RLDKFKLEILKQLEKDIYTK-72 |
| gO6 | 66-EKDIYTKYFNMTRQHIKNLT-85 |
| gO7 | 79-QHIKNLTMNMTEFPRYYILA-98 |
| gO8 | 92-PRYYILAGPIQNNSVTYLWF-111 |
| gO9 | 105-SVTYLWFDFYSTQLRKPAKY-124 |
| gO10 | 118-LRKPAKYVFSEYNHTAKTIT-137 |
| gO11 | 131-HTAKTITFRPPSCGTVPSMT-150 |
| gO12 | 144-GTVPSMTCLSEMLNVSKRND-163 |
| gO13 | 157-NVSKRNDTGEQGCGNFTTFN-176 |
| gO14 | 170-GNFTTFNPMFFNVPRWNTKL-189 |
| gO15 | 183-PRWNTKLYVGSKKVNVDSQT--202 |
| gO16 | 196-VNVDSQTIYFLGLTALLLRY-215 |
| gO17 | 209-TALLLRYAQRNCTHSFYLVN-228 |
| gO18 | 222-HSFYLVNAMSRNLFRVPKYI-241 |
| gO19 | 235-FRVPKYINGTKLKNTMRKLK-254 |
| gO20 | 248-NTMRKLKRKQAPVKEQSEKK-267 |
| gO21 | 261-KEQSEKKSKKSQSTTTPYSP-280 |
| gO22 | 274-TTTPYSPYTTSTALNVTTNA-293 |
| gO23 | 287-LNVTTNATYSVTTTARRVST-306 |
| gO24 | 300-TARRVSTSTIAYRPDSSFMK-319 |
| gO25 | 313-PDSSFMKSIMTTQLRDLATW-332 |
| gO26 | 326-LRDLATWVYTTLRYRQNPFC-345 |
| gO27 | 339-YRQNPFCESSRNRTAVSEFM-358 |
| gO28 | 352-TAVSEFMKNTHVLIRNETPY-371 |
| gO29 | 365-IRNETPYTIYGTLDMSSLYY-384 |
| gO30 | 378-DMSSLYYNETMFVENKTASE-397 |
| gO31 | 391-ENKTASETTPTSPSTGFQRT-410 |
| gO32 | 404-STGFQRTFIDPLWDYLDSLL-423 |
| gO33 | 417-DYLDSLLFLDEIRNFSLQSP-436 |
| gO34 | 430-NFSLQSPTYGNLTPPEHRRA-449 |
| gO35 | 443-PPEHRRAVNLSTLNSLWWWL-462 |

S1 Table: gO peptide library (from strain TR)
