# Supplementary material for: Identification of functionally important domains of human cytomegalovirus gO that act after trimer binding to receptors
Source: PLoS Pathog. 2022 Apr 22;18(4):e1010452. doi: 10.1371/journal.ppat.1010452 (PMC9032346; doi:10.1371/journal.ppat.1010452)
Supplement: S2 Table — Listed are the numbers of the peptide followed by the amino acid sequence and the residue coordinates that define the peptide. (DOCX) [file ppat.1010452.s003.docx]

| gO20-1 | 248-NTMRKLKR-255 |
| --- | --- |
| gO20-2 | 249-TRMKLKRK-256 |
| gO20-3 | 250-MRKLKRKQ-257 |
| gO20-4 | 251-RKLKRKQA-258 |
| gO20-5 | 252-KLKRKQAP-259 |
| gO20-6 | 253-LKRKQAPV-260 |
| gO20-7 | 254-KRKQAPVK-261 |
| gO20-8 | 255-RKQAPVKE-262 |
| gO20-9 | 256-KQAPVKEQ-263 |
| gO20-10 | 257-QAPVKEQS-264 |
| gO20-11 | 258-APVKEQSE-265 |
| gO20-12 | 259-PVKEQSEK-266 |
| gO20-13 | 260-VKEQSEKK-267 |
| gO26-1 | 326-LRDLATWV-333 |
| gO26-2 | 327-RDLATWVY-334 |
| gO26-3 | 328-DLATWVYT-335 |
| gO26-4 | 329-LATWVYTT-336 |
| gO26-5 | 330-ATWVYTTL-337 |
| gO26-6 | 331-TWVYTTLR-338 |
| gO26-7 | 332-WVYTTLRY-339 |
| gO26-8 | 333-VYTTLRYR-340 |
| gO26-9 | 334-YTTLRYRQ-431 |
| gO26-10 | 335-TTLRYRQN-342 |
| gO26-11 | 336-TLRYRQNP-343 |
| gO26-12 | 337-LRYRQNPF-344 |
| gO26-13 | 338-RYRQNPFC-345 |

S2 Table: gO peptides 20-1 to 20-13

and 26-1 to 26-13 (from strain TR)
